# Arterial spin labelling magnetic resonance imaging and perfusion patterns in neurocognitive and other mental disorders: a systematic review

**DOI:** 10.1007/s00234-024-03323-0

**Published:** 2024-03-27

**Authors:** Rita Ferreira, António J. Bastos-Leite

**Affiliations:** Faculty of Medicine, University of Porto, Porto, Portugal

**Keywords:** Arterial spin labeling, Cerebral blood flow, Cognitive impairment, Mental disorders, Perfusion

## Abstract

We reviewed 33 original research studies assessing brain perfusion, using consensus guidelines from a “white paper” issued by the International Society for Magnetic Resonance in Medicine Perfusion Study Group and the European Cooperation in Science and Technology Action BM1103 (“Arterial Spin Labelling Initiative in Dementia”; https://www.cost.eu/actions/BM1103/). The studies were published between 2011 and 2023 and included participants with subjective cognitive decline *plus*; neurocognitive disorders, including mild cognitive impairment (MCI), Alzheimer’s disease (AD), frontotemporal lobar degeneration (FTLD), dementia with Lewy bodies (DLB) and vascular cognitive impairment (VCI); as well as schizophrenia spectrum disorders, bipolar and major depressive disorders, autism spectrum disorder, attention-deficit/hyperactivity disorder, panic disorder and alcohol use disorder. Hypoperfusion associated with cognitive impairment was the major finding across the spectrum of cognitive decline. Regional hyperperfusion also was reported in MCI, AD, frontotemporal dementia phenocopy syndrome and VCI. Hypoperfused structures found to aid in diagnosing AD included the precunei and adjacent posterior cingulate cortices. Hypoperfused structures found to better diagnose patients with FTLD were the anterior cingulate cortices and frontal regions. Hypoperfusion in patients with DLB was found to relatively spare the temporal lobes, even after correction for partial volume effects. Hyperperfusion in the temporal cortices and hypoperfusion in the prefrontal and anterior cingulate cortices were found in patients with schizophrenia, most of whom were on medication and at the chronic stage of illness. Infratentorial structures were found to be abnormally perfused in patients with bipolar or major depressive disorders. Brain perfusion abnormalities were helpful in diagnosing most neurocognitive disorders. Abnormalities reported in VCI and the remaining mental disorders were heterogeneous and not generalisable.

**Supplementary Information:**

The online version contains supplementary material available at 10.1007/s00234-024-03323-0.

## Introduction

Arterial spin labelling (ASL) magnetic resonance imaging (MRI) can obtain absolute quantification of cerebral blood flow (CBF) and assess the corresponding metabolism of the brain. By inverting the longitudinal magnetisation of proton spins as they flow into the brain, ASL uses labelled arterial blood water as a diffusible tracer [1, 2]. Other imaging techniques that assess brain perfusion and metabolism, such as single-photon emission computed tomography (SPECT) and positron emission tomography (PET), rely on exogenous radioactive tracers delivered to (and cleared out of) the brain tissue. Computed tomography and dynamic contrast enhanced (DCE) or dynamic susceptibility contrast (DSC) MRI also can assess perfusion by using a bolus injection of an exogenous contrast agent whose dynamic passage through the brain tissue provides specific perfusion parameters. However, neither SPECT, PET, CT, DCE nor DSC MRI enable absolute quantification of CBF. ASL not only allows for absolute quantification (usually in mL/100 g/min) but also is completely non-invasive because it does not rely on the injection of any exogenous tracer [3–5]. Crucially, ASL is also being used in other organs, apart from the brain [6].

Since its inception in the early 1990s, ASL has greatly improved. Modifications in both MRI sequences and hardware have increased the image quality and reduced the corresponding scanning time [7]. The relatively short acquisition time and simplicity of repeated scanning has been advantageous for follow-up studies, making ASL a useful complement to conventional MRI protocols [3, 4]. To standardise ASL and expand its usage, a consensus statement in the form of a “white paper” issued by the International Society for Magnetic Resonance in Medicine Perfusion Study Group and the European Cooperation in Science and Technology Action BM1103 (“Arterial Spin Labelling Initiative in Dementia”; https://www.cost.eu/actions/BM1103/) was published in 2015 [8]. This consensus recommended the implementation of pseudo-continuous arterial spin labelling (pCASL) with a single post-labelling delay (PLD) time, background suppression and a three-dimensional (3D) readout [8]. This approach helped to standardise both the labelling strategy and MRI sequence acquisition, improve agreement in CBF measurements between studies and facilitate the use of ASL in clinical practice. An updated and expanded consensus on the “current state and guidance on ASL in clinical neuroimaging” was published in 2023 [5].

As the prevalence of neurocognitive disorders (NCD) increases and effective treatments become available, the need for early and accurate clinical diagnoses becomes urgent [9, 10]. Along with NCD like Alzheimer’s disease (AD) and dementia with Lewy bodies (DLB), a high incidence of dementia also occurs in psychosis [11], and psychiatric diagnoses often overlap specific NCD, such as the behavioural variant of frontotemporal dementia (bvFTD) and the corresponding phenocopy syndrome [12]. Many mental disorders are heterogeneous at the pathophysiological and clinical levels [13]. Various symptoms associated with several mental disorders may vary at the level of severity or proportion [14], a concept known as “dimension” (cf. domain) [15]. In the clinical setting, such heterogeneity across symptoms can delay diagnosis.

A growing body of evidence shows that functional brain changes precede structural ones and, hence, physiological parameters may aid in early diagnosis [4, 5, 16]. To the best of our knowledge, no previous study has reported on the clinical application of pCASL in assessing a wide range of mental conditions. For this reason, the purpose of the current review was to compare CBF measurements or detect regions of abnormal brain perfusion, as assessed by pCASL, in patients with neurocognitive or other mental disorders, relative to healthy control (HC) subjects or those with subjective cognitive decline (SCD) [17], as well as to detect brain perfusion abnormalities useful to distinguish patients with different clinical conditions.

## Methods

### Literature search and selection criteria

This review was conducted according to recommendations of the Preferred Reporting Items for Systematic Reviews and Meta-analyses (PRISMA) guidelines [18]. PubMed, WebOfScience and PsycINFO databases were searched for articles published until June 2023 that included terms related to NCD and other mental disorders (i.e. “cognitive disorder”, “cognitive dysfunction”, “cognitive impairment”, “mild neurocognitive disorder”, “mild cognitive disorder”, “mild cognitive impairment”, “dementia”, “major neurocognitive disorder”, “major cognitive disorder”, “mental disorders”, “mental illness”, “psychiatric disorders” or “psychiatric illness”), as well as terms related to imaging and ASL (i.e. “arterial spin labeling”, “arterial spin labelling”, “ASL”, “brain”, “brain imaging” or “neuroimaging”).

Published, peer-reviewed, original research studies on the clinical application of pCASL in patients with mental conditions were eligible for inclusion. Whenever possible, we applied the nomenclature of the *Diagnostic and Statistical Manual of Mental Disorders*, fifth edition (DSM-5) [14]. From an MRI perspective, only pCASL studies using a single PLD time, background suppression and a 3D readout on scanners operating up to 3 Tesla were included, according to the 2015 consensus paper providing technical guidelines for ASL application [8]. Studies also were required to have a comparison group (HC subjects or those with SCD) or to focus on differential diagnosis. Subjects with SCD were defined as individuals with subjective (self-perceived) cognitive complaints worsening over time but without any confirmation on cognitive testing (i.e. a normal objective performance level) [17].

Excluded studies were those not reporting comparisons of CBF measurements in regions of interest (ROI) or not using a voxel-wise approach, studies addressing psychopharmacological or other therapeutic effects only, articles written in languages other than English, review articles (e.g. meta-analyses) and commentaries or editorials on the theoretical background of ASL.

Studies focused only on neurovascular coupling or functional brain connectivity using ASL also were excluded.

### Analysis

All included studies were analysed to determine whether they reported decreased (hypoperfusion), similar or increased brain perfusion (hyperperfusion), relative to HC or SCD subjects or between distinct groups of patients, according to statistical thresholds used in the corresponding publications. When disclosed, we report measures of diagnostic value, such as sensitivity, specificity, positive predictive value and negative predictive value. Likewise, when applied to measure the ability of brain perfusion to distinguish patients from controls or patients with different clinical conditions, we report the area under the receiver operating characteristic (ROC) curve (i.e. the area under the curve [AUC] or summary of the ROC curve)—the integral of a graphical plot illustrating the diagnostic ability of a binary classifier as its discrimination threshold (i.e. cut-off value) varies. An AUC closer to 1.0 indicates better performance of the classifier at distinguishing between two groups. To determine the risk of bias of each study, we also applied a set of 12 predefined questions as a quality assessment tool [19].

## Results

**Fig. 1:**
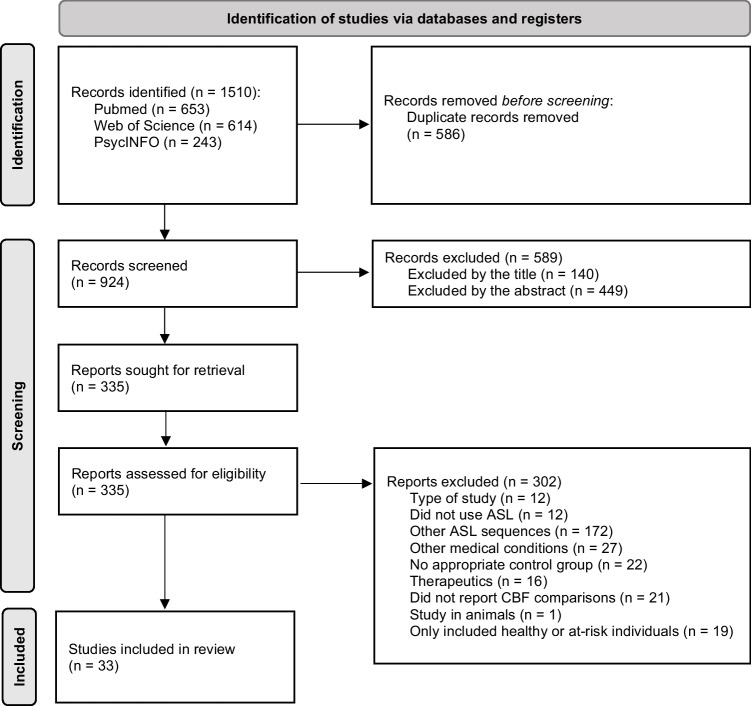
PRISMA flow diagram for search and selection of studies

Figure 1 presents the PRISMA flow diagram for the search and selection of studies. After excluding duplicates, screening titles and abstracts and assessing articles for eligibility, 33 studies published between 2011 and 2023 were included in this review [20–52]. According to the aforementioned quality assessment tool [19], all included studies were rated fair to good. Nineteen (58%) comprised samples of subjects with SCD *plus* and patients with NCD [21–24, 26, 30–32, 34, 35, 37, 38, 43, 46–52], including mild cognitive impairment (MCI), AD, frontotemporal lobar degeneration (FTLD), DLB and vascular cognitive impairment (VCI), according to the corresponding diagnostic criteria [17, 53–63]. Given that subjects with SCD *plus* have “features that increase the likelihood of preclinical AD” [17], we report them as a distinct subgroup belonging to the spectrum of cognitive decline. Four (12%) of the 33 studies encompassed patients with schizophrenia spectrum disorders (SSD) [27, 29, 36, 39], four (12%) comprised patients with major depressive disorder (MDD) [20, 28, 33, 45] and three (9%) comprised patients with bipolar disorders (BD) [33, 40, 45]. Finally, the remaining four (12%) studies included patients with autism spectrum disorder (ASD) [25], attention-deficit/hyperactivity disorder (ADHD) [42], panic disorder [41] and alcohol use disorder [44].

Table 1 summarises findings of the six (18%) studies reporting measures of diagnostic value for brain perfusion, all in the setting of SCD *plus* and NCD [21, 26, 30, 37, 43, 50]. Supplementary Table 1 summarises the main characteristics of all studies included in this review (i.e. demographic data and the PLD time used in each study) [20–52].

**Table 1:** Measures of diagnostic value for pseudo-continuous arterial spin labelling studies on neurocognitive disorders and SCD *plus*

Study	Groups	Brain area	AUC (95% CI)	Cut-off value	Sensitivity; Specificity; PPV; NPV
Binnewijzend et al., 2013 [21]	AD vs SCD	Precuneus and PCC	0.83 (0.76–0.89)	32.9	(80%; 69%; 0.74; 0.71)
Liu et al., 2015 [26]	AD vs HC	PCC	0.89^†^ 0.88^††^	37.8^†^ 36.6^††^	(84.2%; 81.2%; 0.81; 0.84) (84.2%; 81.2%; 0.81; 0.84)
Steketee et al., 2016 [30]	AD vs HC	Precuneus Medial temporal lobe Temporal lobe PCC Thalamus ACC Medial prefrontal cortex	0.85 (0.73–0.97) 0.76 (0.60–0.92) 0.81 (0.67–0.96) 0.84 (0.71–0.97) 0.80 (0.65–0.95) 0.74 (0.56–0.92) 0.74 (0.55–0.92)	31.6 N/A N/A N/A N/A N/A N/A	(77%; 76%; N/A; N/A) N/A N/A N/A N/A N/A N/A
	FTLD vs HC	ACC	0.78 (0.63–0.92)	46.0	(79%; 76%; N/A; N/A)
	AD vs FTLD	PCC	0.74 (0.56–0.92)	46.3	(69%; 68%; N/A; N/A)
Anazodo et al., 2018 [37]	FTLD vs HC	Frontal region Temporal region Insula ACC All ROI	0.78 0.66 0.65 0.76 0.75	N/A N/A N/A N/A N/A	(70%; 70%; N/A; N/A) (60%; 70%; N/A; N/A) (70%; 50%; N/A; N/A) (70%; 80%; N/A; N/A) (80%; 60%; N/A; N/A)
Yang et al., 2021 [43]	SCD *plus* vs HC	Left hippocampus head Left PCC Left precuneus Right PCC Combination of the above	0.71 (0.58–0.84) 0.69 (0.55–0.83) 0.66 (0.55–0.80) 0.65 (0.51–0.79) 0.84 (0.74–0.94)	N/A N/A N/A N/A N/A	N/A N/A N/A N/A N/A
	aMCI vs SCD *plus*	Left precuneus Left thalamus Combination of the above	0.75 (0.63–0.87) 0.69 (0.55–0.83) 0.78 (0.66–0.90)	N/A N/A N/A	N/A N/A N/A
Mao et al., 2023 [50]	AD vs HC	10 temporal-parietal-occipital ROI	0.82	N/A	(81%; 74%; N/A; N/A)
	AD vs FTLD	10 frontal-temporal ROI	0.71	N/A	(81%; 55%; N/A; N/A)

ACC: anterior cingulate cortex, AD: Alzheimer’s disease, aMCI: amnestic mild cognitive impairment, AUC: area under the receiver operating characteristic curve, CI: confidence interval; Cut-off values in mL/100 g/min; FTLD: frontotemporal lobar degeneration; HC: healthy controls; N/A: not applicable; NPV: negative predictive value; PCC: posterior cingulate cortex; PPV: positive predictive value; ROI: regions of interest; SCD: subjective cognitive decline; ^†^Post-labelling delay (PLD) time = 1500 ms; ^††^PLD time = 2500 ms. Unless otherwise specified, the abovementioned brain structures written in the singular form correspond to each side of the midline

### Arterial spin labelling in subjective cognitive decline *plus* and neurocognitive disorders

#### Subjective cognitive decline *plus*

The only study comprising subjects with SCD *plus* found them to have lower values of blood flow in the left hippocampal head and both posterior cingulate cortices (PCC), relative to HC subjects [43]. A positive correlation was found between the left hippocampal head blood flow and auditory verbal learning test delayed recall scores (*r* = 0.386; *p-*value = 0.046). The differentiation between SCD *plus* and HC subjects was best achieved by a combined ROC analysis of CBF values from the left hippocampal head, left precuneus and both PCC, which yielded an AUC of 0.84, but no cut-off value was reported [43].

#### Mild cognitive impairment and Alzheimer’s disease

Table 2 summarises findings of the nine (27%) studies comparing patients with MCI to HC or SCD subjects [21, 24, 34, 35, 43, 47, 48, 50, 52]. Table 3 summarises findings of the 14 (42%) studies comparing patients with AD to HC or SCD subjects [21–24, 26, 30, 32, 35, 38, 47–50, 52]. One study comprised patients under medication (i.e. patients with AD taking acetylcholinesterase inhibitors) [38]. It is plausible that patients in the remaining studies on AD were not taking antidementia drugs.

**Table 2:** Main findings of studies comparing patients with MCI to HC subjects or those with SCD

Binnewijzend et al., 2013 [21]^†^ (MCI vs SCD)	Global cortical, precuneus and PCC, parietal cortices, occipital and frontal cortices CBF ↓*
Ding et al., 2014 [24]^††^ (MCI vs HC)	Hypoperfusion in the inferior temporal cortices, left cuneus, left fusiform gyrus, right middle temporal cortices and right superior parietal lobe, relative to HC subjects*; Hyperperfusion in the frontal lobes and right temporal subcortical white matter, relative to HC subjects*
Dolui et al., 2017 [34]^††^ (MCI vs HC)	Hypoperfusion in the PCC, relative to HC subjects**; Hypoperfusion in the precuneus and hippocampus, relative to HC subjects*
Leeuwis et al., 2017 [35]^†^ (MCI vs SCD)	Global cortical, parietal lobes, occipital lobes, frontal lobes and temporal lobes CBF ↓*
Yang et al., 2021 [43]^†^ (MCI vs HC)	Head of the left hippocampus CBF ↓***; PCC, right thalamus CBF ↓**; Right precuneus, left thalamus, heath of the right hippocampus, head of the left caudate nucleus, white matter of the left occipital and frontal lobes CBF ↓*
Gao et al., 2023 [47]^††^ (MCI vs HC)	Brain perfusion ≈
Camargo et al., 2023 [48]^†^ (MCI vs HC)	Hypoperfusion in the right middle frontal gyrus, relative to HC subjects***; Hyperperfusion in the right superior frontal gyrus, left precuneus and right thalamus, relative to HC subjects***
Mao et al., 2023 [50]^††^ (MCI vs HC)	Right inferior parietal lobule and left superior parietal lobule CBF ↓*
Zhu et al., 2023 [52]^††^ (MCI vs HC)	Brain perfusion ≈

CBF: cerebral blood flow; MCI: mild cognitive impairment; PCC: posterior cingulate cortex; SCD: subjective cognitive decline; ↓: decreased, relative to healthy control (HC) or SCD subjects; ≈: no significant difference between groups; ^†^With correction for partial volume effects; ^††^Without correction for partial volume effects (or not reported); *p-value ≤ .05; **p-value ≤ .01; ***p-value ≤ .001. Unless otherwise specified, the abovementioned brain structures written in the singular form correspond to each side of the midline

**Table 3:** Main findings of studies comparing patients with AD to HC subjects or those with SCD

Binnewijzend et al., 2013 [21]^†^ (AD vs SCD)	Global cortical and white matter, precuneus and PCC, parietal cortices, occipital cortices, temporal cortices and frontal cortices CBF ↓***
Benedictus et al., 2014 [22]^†^ (AD vs SCD)	Global cortical CBF ↓**
Binnewijzend et al., 2014 [23]^†^ (AD vs SCD)	Global cortical, precuneus and PCC, parietal cortices and occipital cortices CBF ↓***; Temporal cortices and frontal cortices CBF ↓**
Ding et al., 2014 [24]^††^ (AD vs HC)	Hypoperfusion in the parietal-occipital cortices, right fusiform gyrus and left side of the splenium of the corpus callosum, relative to HC subjects*; Hyperperfusion in the thalami, right paracentral lobule, white matter of the right temporal lobe, right caudate nucleus and right putamen, relative to HC subjects*
Liu et al., 2015 [26]^††^ (AD vs HC)	Temporal lobes, middle cingulate gyri, precuneus and PCC, left inferior parietal gyrus, left angular gyrus and left superior frontal gyrus CBF ↓***
Steketee et al., 2016 [30]^†^ (AD vs HC)	Precuneus and PCC CBF ↓***; Supratentorial cortical, thalami, temporal lobes and occipital lobes CBF ↓**; Medial PFC, ACC, calcarine cortices and medial temporal lobes CBF ↓*
Steketee et al., 2016 [32]^†^ (AD vs HC)	Hypoperfusion in the posterior part of the temporal lobes, left superior temporal gyrus, precunei and left PCC and lateral part of the right occipital lobe, relative to HC subjects*
Leeuwis et al., 2017 [35]^†^ (AD vs SCD)	Global cortical, parietal lobes, occipital lobes, frontal lobes and temporal lobes CBF ↓*
Huang et al., 2018 [38]^†^ (AD vs HC)	Inferior temporal lobes, posterior parietal cortices, angular gyri, precuneus, PCC and middle prefrontal cortices CBF ↓*
Gao et al., 2023 [47]^††^ (AD vs HC)	Hypoperfusion in the middle and inferior temporal and occipital gyri, middle frontal gyri, right superior frontal gyrus and right insula, relative to HC subjects*; Hyperperfusion in the supplementary motor areas, left precentral gyrus, left postcentral gyrus and putamina, relative to HC subjects*
Camargo et al., 2023 [48]^†^ (AD vs HC)	Hypoperfusion in the cerebellum, left putamen, left insula, temporal lobes and orbital gyri, relative to HC subjects***
Dong et al., 2023 [49]^††^ (AD vs HC)	Hypoperfusion in the precuneus and PCC, angular gyri, parietal and temporal lobes, relative to HC subjects*
Mao et al., 2023 [50]^††^ (AD vs HC)	Global cortical (especially in the temporal-parietal-occipital regions) CBF ↓* ^to^ ***
Zhu et al., 2023 [52]^††^ (AD vs HC)	Hypoperfusion in the left medial temporal lobe, left PCC and angular gyri, relative to HC subjects*; Hyperperfusion in the left thalamus and left middle cingulate cortex, relative to HC subjects*

ACC: anterior cingulate cortex; AD: Alzheimer’s disease; CBF: cerebral blood flow; PCC: posterior cingulate cortex; PFC: prefrontal cortex; SCD: subjective cognitive decline. ↓: decreased, relative to healthy control (HC) or SCD subjects; ^†^With correction for partial volume effects; ^††^Without correction for partial volume effects (or not reported); *p-value ≤ .05; **p-value ≤ .01; ***p-value ≤ .001. Unless otherwise specified, the abovementioned brain structures written in the singular form correspond to each side of the midline

Global (supratentorial) cortical hypoperfusion was found in patients with MCI and those with AD [21–23, 30, 35, 50]. One study compared patients with AD and HC subjects using two single PLD times (1500 and 2500 ms). In both circumstances, the same clusters of hypoperfusion were found in patients with AD, but the number of hypoperfused voxels was lower when using the longer PLD time. This particular difference was attributed to compensatory phenomena caused by the surrounding structures [26].

Some studies reported hyperperfusion in the frontal lobes, left precuneus, right thalamus and right temporal white matter of patients with MCI [24, 48]. Regional hyperperfusion also was reported in the thalami [24, 52], right paracentral lobule, white matter of the right temporal lobe, right striatum [24], supplementary motor areas, putamina, left somatosensory cortex [47] and left middle cingulate cortex of patients with AD [52], relative to control subjects. Hyperperfusion was attributed to compensatory phenomena counteracting pathophysiology.

Studies comprising patients with MCI reported global cortical [21] and regional hypoperfusion in the parietal and occipital lobes [21, 24, 35, 43, 50], both PCC [21, 34, 43] and the adjacent precunei (which partially overlap and border the PCC [64]) [34, 43], as well as in the frontal lobes [21, 35, 43, 48], temporal lobes [24, 35] and hippocampi [34, 43]. Although two studies comprising patients with MCI did not find a significant difference in brain perfusion between these patients and HC subjects [47, 52], a statistical interaction revealed that altered perfusion in the right hippocampus was influenced by the number of years of education (*p-*value corrected for multiple comparisons < 0.05) [52].

**Fig. 2:**
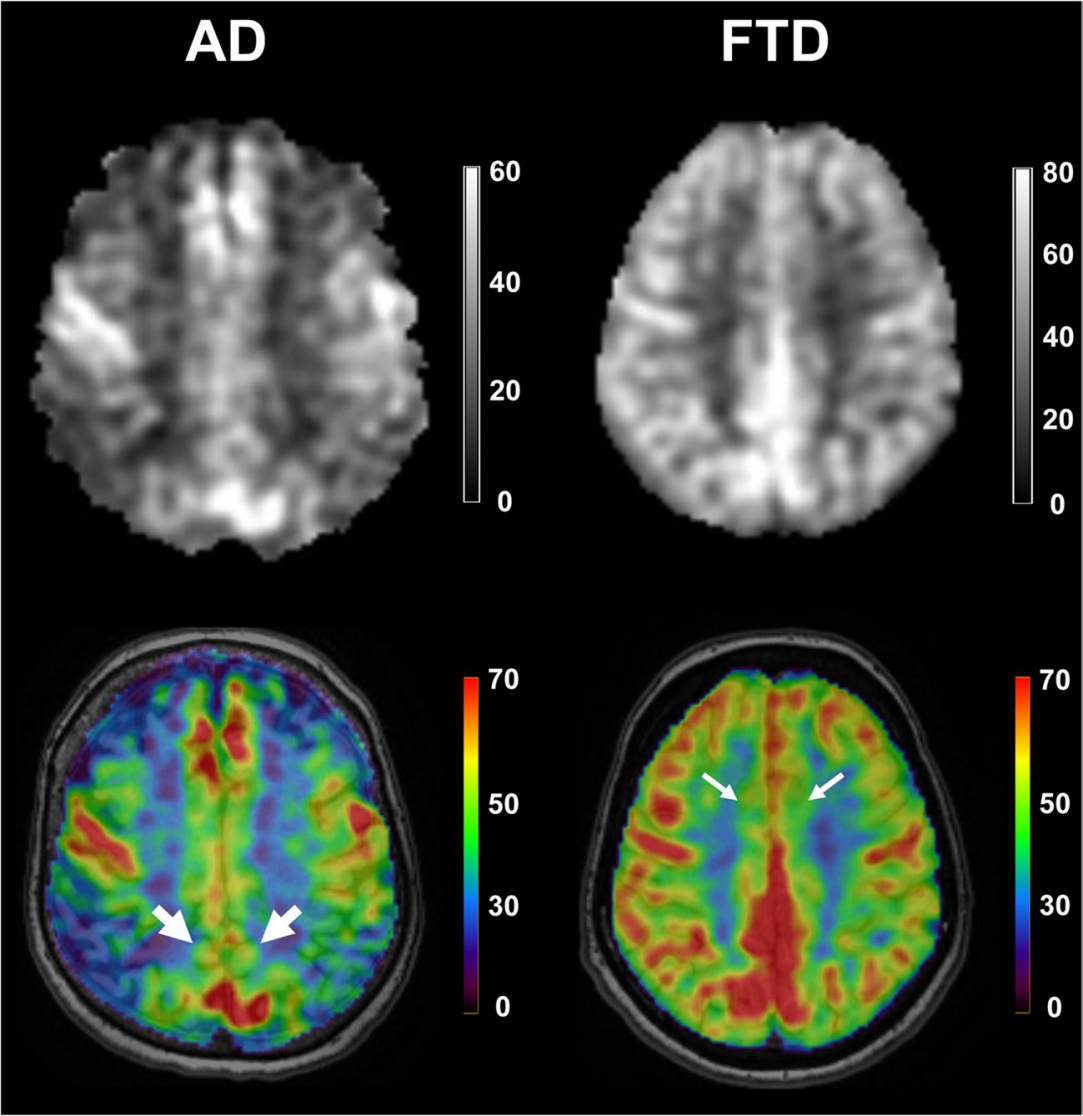
Cerebral blood flow maps of one patient with Alzheimer’s disease (AD, left column) and one patient with frontotemporal dementia (FTD, right column). The bottom row shows colour-coded cerebral blood flow maps coregistered onto high-resolution T1-weighted magnetic resonance images. Please note the occurrence of prominent hypoperfusion in the precunei and adjacent posterior cingulate cortices (thick arrows) of the patient with AD, as well as focal hypoperfusion in the anterior cingulate cortices of the patient with FTD (thin arrows). Please also note that the grey scale, not the colour scale, is different between patients to optimise the display of relative hypoperfusion. Reproduced under the Creative Commons Attribution 4.0 International License with permission of the authors: Steketee RME, Bron EE, Meijboom R, Houston GC, Klein S, Mutsaerts HJMM, Mendez Orellana CP, de Jong FJ, van Swieten JC, van der Lugt A, Smits M (2016) Early-stage differentiation between presenile Alzheimer's disease and frontotemporal dementia using arterial spin labeling MRI. Eur Radiol 26(1):244–253 [30]

In patients with AD, 13 studies found hypoperfusion in the temporal lobes [21, 23, 24, 26, 30, 32, 35, 38, 47–50, 52], nine in the precunei and cingulate cortices [21, 23, 26, 30, 32, 38, 49, 50, 52] and nine in the parietal lobes [21, 23, 24, 26, 35, 38, 49, 50, 52]. The occipital [21, 23, 24, 30, 32, 35, 50] and frontal [21, 22, 26, 30, 35, 38, 48, 50] lobes also were found to be hypoperfused in AD. The most prominent changes were observed in the precunei, PCC and parietal regions [21, 23]. The hypoperfused structures found to aid in diagnosing patients with AD were the precunei and adjacent PCC (Fig. 2). ROC curves generated for CBF measurements in these regions revealed optimal cut-off values ranging from 31.6 to 37.8 ml/100 g/min to differentiate patients with AD from HC subjects or those with SCD (sensitivity: 77–84%; specificity: 69–81%; AUC: 0.83–0.89) [21, 26, 30].

One study by Benedictus et al. investigated the associations of normalised brain volumes and white matter hyperintensities volumes with CBF. It found that both smaller normalised brain volumes and larger white matter hyperintensities volumes were independently associated with lower cortical CBF in patients with AD, especially after statistical adjustment for hippocampal volume and concomitance of microbleeds and lacunar infarcts [22].

Six studies reported significant associations between brain perfusion and cognitive impairment [21, 26, 35, 38, 48, 49]. Three [21, 26, 49] used the Mini Mental State Examination (MMSE) [65]. One [38] used the Cognitive Ability Screening Instrument [66]. One [35] used separate measures to assess various cognitive domains (i.e. memory, attention, executive and visuospatial functioning, as well as language). Another [48] used the Wechsler memory scale—revised immediate-recall subtest [67]. All but one [48] of these studies demonstrated lower values of brain perfusion to be associated with worse cognitive performance in MCI and AD, mainly driven by strong correlations between lower CBF values and cognitive impairment in AD [21]. Patients with AD were found to have lower MMSE scores and decreased CBF, especially in the parietal lobes, precunei and middle and posterior cingulate cortices [21, 26]. Specifically, Binnewijzend et al. found a strong association between MMSE scores and CBF values [21]. A standardised β value of 0.48 (*p-*value ≤ 0.001) was found for the association between MMSE scores and CBF, corrected for partial volume effects, in the parietal lobes, precunei and adjacent PCC. The corresponding value of 0.42 (*p-*value ≤ 0.001) was found for the association between MMSE scores and global brain CBF [21]. A study by Liu et al. confirmed a similar association involving the left posterior cingulate cortex at a PLD time of 1500 ms (*r* = 0.628; *p-*value ≤ 0.001), but there was no correction for partial volume effects [26]. Finally, a study by Dong et al. found the left middle temporal gyrus perfusion to be positively correlated with MMSE scores (*p-*value corrected for multiple comparisons < 0.05) [49].

#### Frontotemporal lobar degeneration

Table 4 summarises findings of the six (18%) studies comparing patients with FTLD to HC or SCD subjects [23, 30–32, 37, 46]. Most patients had bvFTD, and some had other FTLD subtypes [30, 37, 46]. One study included a subgroup of seven patients with the following diagnostic criteria for phenocopy frontotemporal dementia (phFTD): presence of behavioural symptoms, imaging findings not consistent with bvFTD, no progression within (at least) one year after diagnosis, no C9ORF72 mutations and no other concurrent identifiable mental disorder. This study found hyperperfusion in the subcallosal areas of these seven patients, relative to HC subjects [31].

**Table 4:** Main findings of studies comparing patients with FTLD to HC subjects or those with SCD

Binnewijzend et al., 2014 [23]^†^	Global cortical, precuneus and PCC, occipital cortex CBF ↓***;
(bvFTD vs SCD)	Parietal cortices, frontal cortices and cerebellum CBF ↓**
Steketee et al., 2016 [30]^†^	
(FTLD vs HC)	ACC CBF ↓*
Steketee et al., 2016 [31]^†^	
(bvFTD vs HC)	Left straight gyrus, left inferior frontal gyrus, left orbitofrontal gyrus CBF ↓*
Steketee et al., 2016 [32]^†^	
(bvFTD vs HC)	Brain perfusion ≈
Anazodo et al., 2018 [37]^†^	
(FTLD vs HC)	Hypoperfusion in the ACC, superior frontal gyri, medial frontal gyri, *pars opercularis* of the right inferior frontal gyrus, *pars triangularis* of the right inferior frontal gyrus, right precentral gyrus, right superior temporal gyrus, right middle temporal gyrus, right superior and middle temporal poles, relative to HC subjects*
Ssali et al., 2022 [46]^††^	
(bvFTD vs HC)	Frontal poles, superior and middle frontal gyri, inferior and anterior temporal poles, ACC and insula CBF ↓*
(SD vs HC)	Predominantly left brain hemispheric (temporal pole, middle temporal gyrus, inferior temporal gyrus and fusiform gyrus) CBF ↓*
(PNFA vs HC)	Predominantly left brain hemispheric (temporal pole, inferior temporal gyrus, insula, frontal pole and left perisylvian region) CBF ↓*

ACC: anterior cingulate cortex; bvFTD: behavioural variant of frontotemporal dementia; CBF: cerebral blood flow; FTLD: frontotemporal lobar degeneration; PCC: posterior cingulate cortex; PNFA: primary progressive nonfluent aphasia; SCD: subjective cognitive decline; SD: semantic dementia; ↓: decreased, relative to healthy control (HC) or SCD subjects; ≈: no significant difference between groups ^†^With correction for partial volume effects; ^††^Without correction for partial volume effects (or not reported); *p-value ≤ .05; **p-value ≤ .01; ***p-value ≤ .001. Unless otherwise specified, the abovementioned brain structures written in the singular form correspond to each side of the midline

Patients with FTLD also were found to have lower CBF in the anterior cingulate cortices (ACC) and the frontal lobes [30, 37, 46]. One study found patients with semantic dementia and primary progressive nonfluent aphasia to have hypoperfusion predominantly in left brain hemispheric structures [46], but the sample size was small. Finally, Steketee et al. did not find a significant brain perfusion difference between patients with bvFTD and HC subjects [32].

The hypoperfused structures found to better help diagnose patients with FTLD were the ACC and frontal regions (Fig. 2). In fact, a ROC analysis yielded an optimal cut-off value of 46 ml/100 g/min for CBF in the ACC to differentiate patients with FTLD from HC subjects (sensitivity = 79%; specificity = 76%; AUC = 0.78) [30]. Slightly lower diagnostic values were found in another study (Table 1) that did not report any cut-off value [37].

#### Dementia with Lewy bodies

One study compared patients with DLB to subjects with SCD [23]. The study found a pattern of global cortical hypoperfusion in patients with DLB involving the frontal, parietal and occipital cortices, the precunei and adjacent PCC, as well as the cerebellum. Despite correction for partial volume effects, a relative sparing of perfusion in the temporal lobes was demonstrated [23], a finding mirroring the well-known structural sparing of these regions in DLB.

#### Vascular cognitive impairment

One study compared brain perfusion between patients with VCI and HC subjects [51]. Compared to the latter, patients with small vessel VCI were found to have hypoperfusion in the right middle frontal and superior temporal gyri. Patients with large vessel VCI were found to have hypoperfusion in the left inferior parietal, left inferior frontal and left middle frontal gyri. Conversely, patients with small vessel VCI were found to have hyperperfusion in the right hippocampus, cerebellar vermis and left middle and posterior cingulate cortices, whereas hyperperfusion was found in the right thalamus of patients with large vessel VCI. In addition, this study found decreased neurovascular coupling at high levels of the cortical hierarchy involved in cognitive control and emotion regulation [51].

### Differential diagnosis of neurocognitive disorders

#### Mild cognitive impairment versus subjective cognitive decline *plus*

Patients with MCI were found to have lower CBF in the left precuneus and left thalamus, relative to subjects with SCD *plus*. Differentiating subjects with SCD *plus* from patients with MCI was best accomplished by combining CBF values from the left precuneus and thalamus whose ROC analysis yielded an AUC of 0.78, but no cut-off value was reported [43].

#### Alzheimer’s disease versus mild cognitive impairment

Table 5 summarises findings of the seven (21%) studies comparing patients with AD to those with MCI [21, 24, 35, 47, 48, 50, 52]. Even though patients with MCI or AD were found to have a similar hypoperfusion pattern, absolute CBF values were lower in those with AD [21, 35]. Specifically, hypoperfusion was found in the parietal [21, 35, 48, 50, 52], temporal [21, 47, 50, 52], frontal [35, 47, 48] and occipital [35, 50] lobes. Moreover, the precunei [21, 47] and adjacent PCC [21, 48], left posterior cingulate cortex [50, 52] and left anterior cingulate cortex [24] were further hypoperfused in patients with AD. Contrary to the abovementioned findings, hyperperfusion was reported in various regions of the right brain hemisphere [24], supplementary motor areas, striata and left precentral gyrus [47, 48], as well as in the orbitofrontal cortices, temporal poles, medial temporal lobes, both ACC, cerebellum [48] and in the left thalamus [52] of patients with AD, relative to patients with MCI.

**Table 5:** Main findings of studies comparing patients with Alzheimer’s disease to those with mild cognitive impairment

Binnewijzend et al., 2013 [21]^†^	White matter CBF ↓**; Parietal cortices, precuneus and PCC, temporal cortex CBF ↓*
Ding et al., 2014 [24]^††^	Hypoperfusion in the left medial frontal lobe, left parietal cortex, right middle temporal-occipital lobe and left side of the anterior cingulate gyrus, relative to patients with mild cognitive impairment*; Hyperperfusion in the right medial frontal gyrus, right paracentral lobule, right limbic lobe (including the PCC, cuneus, fusiform and the parahippocampal gyrus), right putamen, right caudate and lentiform nuclei and right thalamus, relative to patients with mild cognitive impairment*
Leeuwis et al., 2017 [35]^†^	Global cortical, parietal lobes, occipital lobes and frontal lobes CBF ↓*
Gao et al., 2022 [47]^††^	Hypoperfusion in the middle and inferior temporal gyri, precunei, right middle frontal gyrus and left middle and inferior frontal gyri, relative to patients with mild cognitive impairment*; Hyperperfusion in the supplementary motor areas, left precentral gyrus, putamina and caudate nuclei, relative to patients with mild cognitive impairment*
Camargo et al., 2023 [48]^†^	Hypoperfusion in the prefrontal cortices, caudate nuclei, PCC and parietal cortices, relative to patients with mild cognitive impairment*** Hyperperfusion in the orbitofrontal cortices, striata, temporal poles, medial temporal lobes, ACC and cerebellum, relative to patients with mild cognitive impairment***
Mao et al., 2023 [50]^††^	Left PCC, angular gyri, right middle occipital gyrus, right temporal pole and inferior occipital gyri CBF ↓*
Zhu et al., 2023 [52]^††^	Hypoperfusion in the left middle temporal gyrus, left PCC and angular gyri, relative to patients with mild cognitive impairment* Hyperperfusion in the left thalamus, relative to patients with mild cognitive impairment*

CBF: cerebral blood flow; PCC: posterior cingulate cortex; ↓: decreased, relative to patients with mild cognitive impairment; †With correction for partial volume effects; ††Without correction for partial volume effects (or not reported); *p-value ≤ .05; **p-value ≤ .01; ***p-value ≤ .001. Unless otherwise specified, the abovementioned brain structures written in the singular form correspond to each side of the midline

#### Alzheimer’s disease versus frontotemporal lobar degeneration

Table 6 summarises findings of the four (12%) studies comparing patients with AD to those with FTLD [23, 30, 32, 50]. Despite some overlapping areas of hypoperfusion, all but one [50] of these studies found lower perfusion in the temporal cortices (i.e. right hippocampus, left superior and right inferior temporal gyri and right fusiform gyrus) [23, 32], both PCC [30] and orbitofrontal gyri [32] in patients with AD, relative to patients with FTLD. A ROC curve generated for CBF measurements in the PCC revealed an optimal cut-off value of 46.3 ml/100 g/min to differentiate patients with AD from those with FTLD (sensitivity = 69%; specificity = 68%; AUC = 0.74) [30].

**Table 6:** Main findings of studies comparing patients with Alzheimer’s disease to those with frontotemporal lobar degeneration

Binnewijzend et al., 2014 [23]^†^ (AD vs bvFTD)	Temporal cortex CBF ↓**
Steketee et al., 2016 [30]^†^ (AD vs FTLD)	PCC CBF ↓*
Steketee et al., 2016 [32]^†^ (AD vs bvFTD)	Hypoperfusion in the orbitofrontal gyri, right hippocampal formation, left superior and right inferior temporal gyri and right fusiform gyrus, relative to patients with behavioural variant of frontotemporal dementia*
Mao et al., 2023 [50]^††^ (AD vs FTLD)	Left temporal pole, left amygdala, left insula, left caudate nucleus and left middle cingulate gyrus; most frontal gyri, ACC, hippocampus, parahippocampal gyri and entorhinal areas CBF ↑* ^to^ **

bvFTD: behavioural variant of frontotemporal dementia; CBF: cerebral blood flow; PCC: posterior cingulate cortex; ↓: decreased, relative to patients with bvFTD or other frontotemporal lobar degeneration (FTLD) subtypes; ↑: increased, relative to patients with FTLD; ^†^With correction for partial volume effects; ^††^Without correction for partial volume effects (or not reported); *p-value ≤ .05; **p-value ≤ .01. Unless otherwise specified, the abovementioned brain structures written in the singular form correspond to each side of the midline

#### Behavioural variant of frontotemporal dementia versus its phenocopy syndrome

Steketee et al. compared CBF values between patients with bvFTD and those with phFTD [31]. The subcallosal areas were found to be hyperperfused in patients with phFTD, relative to patients with bvFTD. This relative hyperperfusion may reflect compensatory phenomena counteracting incipient pathophysiology. Patients with bvFTD showed lower CBF in both straight gyri, the left superior and inferior frontal gyri and the left orbitofrontal gyrus, relative to patients with phFTD [31].

#### Alzheimer’s disease versus dementia with Lewy bodies

The study comprising patients with DLB also compared CBF values between these patients and those with AD. The ROI analysis showed more cortical hypoperfusion among patients with DLB, especially in the frontal, parietal and occipital cortices, as well as in the precunei and adjacent PCC [23].

### Arterial spin labelling in psychiatric disorders

#### Schizophrenia spectrum disorders

Table 7 summarises findings of the four (12%) studies comparing brain perfusion between patients with SSD and HC subjects [27, 29, 36, 39]. Most patients had chronic symptoms (approximate mean duration of illness ≥ 10 years) and were on antipsychotic medication. In patients with SSD, hyperperfusion was found bilaterally in the temporal cortices [27, 29, 36], striata, thalami [27, 29, 36] and sensorimotor cortices [29, 36]. Furthermore, whereas the middle cingulate cortices were found to be hyperperfused in SSD [36], hypoperfusion was documented in the prefrontal cortices [29, 36, 39], ACC [27, 29, 36], occipital gyri [27, 29, 36], insular cortices [29, 36], parietal cortices [29, 39] and supplementary motor areas [36].

**Table 7:** Main findings of studies comparing patients with schizophrenia spectrum disorders to healthy control subjects

Zhu et al., 2015 [27]^†^ (Patients with schizophrenia)	Hyperperfusion in the inferior temporal gyri, thalami, putamina and left middle temporal gyrus, relative to healthy control subjects*; Hypoperfusion in ACC, middle occipital gyri and left middle frontal gyrus, relative to healthy control subjects*
Ma et al., 2016 [29]^††^ (Patients with schizophrenia)	Hyperperfusion in the temporal cortices, sensorimotor cortices, basal ganglia and thalami, relative to healthy control subjects^§^*; Hypoperfusion in the medial prefrontal cortices, ACC and insula, dorsolateral prefrontal cortices, occipital^§^ and parietal^§^ cortices, relative to healthy control subjects*
Zhu et al., 2017 [36]^†^ (Patients with schizophrenia)	Hyperperfusion in the temporal cortices, sensorimotor cortices, middle cingulate cortices, striata and thalami, relative to healthy control subjects*; Global cortical hypoperfusion, relative to healthy control subjects**; Hypoperfusion in the prefrontal cortices and ACC, occipital cortices, insula and supplementary motor area, relative to healthy control subjects*
Kim et al., 2019 [39]^††^ (Patients with SSD)	Hypoperfusion in the dorsolateral prefrontal cortex, relative to healthy control subjects**; Hypoperfusion in the posterior parietal cortex, relative to healthy control subjects*

ACC: anterior cingulate cortex; SSD: schizophrenia spectrum disorders; ^§^differences in perfusion only found in the subgroup of female patients versus healthy female subjects. ^†^With correction for partial volume effects; ^††^Without correction for partial volume effects (or not reported); *p-value ≤ .05; **p-value ≤ .01. Unless otherwise specified, the abovementioned brain structures written in the singular form correspond to each side of the midline

Two of the abovementioned studies [27, 29] reported associations between brain perfusion and Positive and Negative Syndrome Scale (PANSS) [68] scores. A positive correlation was found between perfusion in the left middle temporal gyrus and PANSS positive scores in women (partial *r*_*s*_ = 0.344, *p-*value = 0.026) [29]. There was also a positive correlation between perfusion in the left inferior temporal gyrus and PANSS negative scores (*r* = 0.261, *p-*value = 0.009) [27]. Finally, a negative correlation was found between insular perfusion and PANSS negative scores (*r* = -0.238, *p-*value = 0.018) [27].

#### Bipolar disorders

Table 8 summarises findings of the three (9%) studies comparing brain perfusion between patients with BD and HC subjects [33, 40, 45]. Across the studies, participants had distinct characteristics. Specifically, one study [33] did not specify the type of bipolar disorder, one [40] included only patients with bipolar I disorder (i.e. with at least one manic episode) and one [45] comprised only patients with bipolar II disorder (i.e. with hypomanic and depressive episodes). Two of these three studies included only patients with BD experiencing a depressive episode [33, 45]. Participants in the remaining study [40] were under the effects of medication and clinically stable at the time of assessment. All participants of one study [45] were either drug naïve or unmedicated for at least six months.

**Table 8:** Main findings of studies comparing patients with bipolar disorders to healthy control subjects

Zhao et al., 2016 [33]^††^	Left dentate nucleus of the cerebellum CBF ↓*
Dai et al., 2020[40]^††^	Increased perfusion fluctuations in the left fusiform gyrus and adjacent inferior temporal region, relative to healthy control subjects*
Chen et al., 2022 [45]^††^	Hyperperfusion on the left side of the posterior lobe of the cerebellum and left middle temporal gyrus, relative to healthy control subjects*

CBF: cerebral blood flow; ↓: decreased, relative to healthy control subjects; ^††^CBF uncorrected for partial volume effects (or not reported); *p-value ≤ .05

The study by Zhao et al. found decreased CBF in the left dentate nucleus of the cerebellum in patients with BD, relative to HC subjects [33]. The study by Dai et al. used a dynamic pCASL approach and found increased perfusion fluctuations in the left fusiform gyrus and adjacent inferior temporal region of patients with bipolar I disorder, relative to HC subjects [40]. Additionally, a marginally significant increase in perfusion fluctuations was documented in the right temporal pole and adjacent inferior temporal region (*p-*value corrected for multiple comparisons = 0.063) [40]. The study by Chen et al. found hyperperfusion on the left side of the posterior lobe of the cerebellum (neocerebellum) and in the left middle temporal gyrus of patients with bipolar II disorder, relative to HC subjects [45].

#### Major depressive disorder

Table 9 summarises findings of the four (12%) studies comparing patients with MDD to HC subjects [20, 28, 33, 45]. All patients were experiencing a depressive episode at the time of assessment. Their average scores on the Hamilton Depression Rating Scale (HDRS) [69] indicated at least moderate severity of depression. Regarding medication, one study [20] comprised 65% of patients on antidepressant or antipsychotic medication, another [33] comprised 23% of patients on antidepressants, and two [28, 45] had no patients taking any relevant medication.

**Table 9:** Main findings of studies comparing patients with major depressive disorder to healthy control subjects

Järnum et al., 2011 [20]^††^	Baseline CBF ≈
Kaichi et al., 2016 [28]^††^	Hyperperfusion in the left inferior parietal gyrus, left middle and inferior temporal gyri; left superior, middle and inferior frontal gyri, left insula and left anterior cingulate cortex, relative to healthy control subjects**; Hypoperfusion in the right lingual gyrus and right superior temporal gyrus, relative to healthy control subjects**
Zhao et al., 2016 [33]^††^	Superior cerebellar peduncles and left dentate nucleus of the cerebellum CBF ↓*
Chen et al., 2022 [45]^††^	Hyperperfusion on the left side of the posterior lobe of cerebellum and left middle temporal gyrus, relative to healthy control subjects*

CBF: cerebral blood flow; ↓: decreased, relative to healthy control subjects; ≈: no significant difference between groups; ^††^CBF uncorrected for partial volume effects (or not reported); *p-value ≤ .05; **p-value ≤ .01 (uncorrected)

The study by Järnum et al. did not find significant CBF differences between patients with MDD and HC subjects [20]. However, this study conducted a subgroup analysis, dividing patients with MDD into remitting (n = 7) and non-remitting (n = 6) ones, depending on whether they did or did not have an HRDS-17 score ≤ 7 after a 6-month follow-up period on medication, respectively. At baseline, non-remitting patients with MDD showed significantly lower CBF in the frontal grey and white matter, both ACC, as well as in the temporal and parietal cortices, relative to HC subjects. Remitting patients with MDD showed no CBF differences, relative to HC subjects, but were found to have higher CBF in the parietal white matter (*p-*value = 0.04), relative to non-remitting patients [20]. The study by Kaichi et al. found hyperperfusion in various cortical regions of the left brain hemisphere. It also found hypoperfusion in the right lingual gyrus and the right superior temporal gyrus of patients [28]. The study by Zhao et al. found decreased CBF in patients with MDD at the superior cerebellar peduncles and the left dentate nucleus of the cerebellum [33]. The study by Chen et al. documented hyperperfusion in patients with MDD on the left side of the neocerebellum and in the left middle temporal gyrus, the latter significantly correlated with HDRS scores (*r* = 0.322, *p-*value = 0.002) [45].

#### Bipolar and major depressive disorders

Two (6%) of the included studies in this review assessed brain perfusion in samples of patients either with BD or MDD during a depressive episode [33, 45]. In the study by Zhao et al., decreased CBF in the left dentate nucleus of the cerebellum was found both in BD and MDD [33]. In the study by Chen et al., both patients with unmedicated bipolar II disorder and those with MDD had hyperperfusion on the left side of the neocerebellum and in the left middle temporal gyrus [45].

#### Other psychiatric disorders

One study on ASD compared brain perfusion between high-functioning autist children/adolescents and HC subjects matched for age, gender and intelligence quotient [25]. On average, patients with high-functioning autism scored 7.7 on the Autism Diagnostic Observation Schedule [70] severity scale, and had a Social Responsiveness Scale [71] total t score of 74.7. At the time of assessment, 41% of patients were receiving medication. A voxel-wise comparison demonstrated a pattern of widespread hyperperfusion at the frontotemporal regions, especially in the medial orbitofrontal cortices and both inferior frontal opercula, as well as in the left middle and inferior temporal gyri and the right precentral gyrus of patients with ASD, relative to HC subjects. By contrast, hypoperfusion was documented in the ACC of patients with ASD. Although this study found increased functional connectivity in the anterior module of the default mode network in patients, there was hypoperfusion in the same region [25].

One study on ADHD compared brain perfusion between patients with this condition and HC subjects [42]. Most (78%) patients had a predominantly inattentive presentation of ADHD. The remaining 22% had a combined presentation of ADHD symptoms. Widespread hypoperfusion was found in patients with ADHD in the left cerebral hemisphere, especially in the left insula, orbitofrontal cortex, putamen, globus pallidus, amygdala, supramarginal gyrus, Rolandic operculum, left temporal gyri, hippocampus, parahippocampal gyrus and left olfactory gyrus (*p-*value corrected for multiple comparisons < 0.05) [42].

One study on panic disorder compared brain perfusion between the afflicted patients and HC subjects [41]. Patients included in this study had at least one panic attack and were drug-free for at least two weeks prior to assessment. They had an average score of 13.2 on the Hamilton Rating Scale for Anxiety [72] and 9.7 on the Panic Disorder Severity Scale [73]. There was significant hypoperfusion and cortical thinning in the right fusiform gyrus in patients with panic disorder, relative to HC subjects. Furthermore, in patients with panic disorder, Z-scores calculated from perfusion values of the right fusiform gyrus were found to be negatively correlated both with the Hamilton Rating Scale for Anxiety (*r* = -0.512; *p-*value = 0.007) and Panic Disorder Severity Scale (*r* = -0.521; *p-*value = 0.006) scores [41].

Finally, one study on alcohol use disorder compared perfusion in the salience network of patients, relative to HC subjects (“social drinking controls”) [44]. Patients consumed an average of 30 drinks per week and had an average Alcohol Use Disorders Identification Test [74] score of 13.1. Patients with alcohol use disorder were found to have hypoperfusion in the insular cortices, relative to HC subjects. Furthermore, a negative correlation was found between perfusion in the ACC and Alcohol Use Disorders Identification Test scores (*p-*value corrected for multiple comparisons = 0.04), as well as between perfusion in the left frontal operculum and the number of drinks consumed per week (*p-*value corrected for multiple comparisons = 0.01) [44].

## Discussion

Most studies included in this review comprised subjects with cognitive impairment, and most reported either global or regional brain hypoperfusion in subjects with SCD *plus* or patients with MCI, AD, FTLD, DLB or VCI, relative to HC or SCD subjects [21–24, 26, 30, 32, 34, 35, 38, 43, 47–52]. We also found reports of regional hyperperfusion in MCI [24], AD [47], phFTD [31] and VCI [51], mostly attributed to compensatory phenomena counteracting pathophysiology. Global cortical hypoperfusion was found either in patients with MCI or AD. Furthermore, hypoperfusion in the precunei and adjacent PCC was characteristic of patients with AD [21–24, 26, 30, 32, 35, 38, 47, 49, 52]. Crucially and as previously mentioned in the section of results, ROC curves generated for CBF measurements in these regions revealed optimal cut-off values, ranging from 31.6 to 37.8 ml/100 g/min, to diagnose patients with AD [21, 26, 30].

Apart from differentiating patients with AD from control subjects, a major finding of the current review was that brain hypoperfusion, as determined by pCASL, enables early detection of AD and thus serves as a biomarker of incipient cognitive impairment. Moreover, pCASL helps to differentiate subjects across the spectrum of cognitive decline, ranging from HC subjects to patients with AD [21, 35, 43]. In fact, a more pronounced hypoperfusion was documented in AD, relative to MCI [21, 35]; in MCI, relative to SCD *plus* [43]; and in SCD *plus*, relative HC subjects [43]. These findings support the notion that CBF could be incorporated into models of AD staging as an early biomarker [4, 35]. Furthermore, lower values of both global and regional CBF were found to be associated with the severity of cognitive impairment in SCD *plus*, MCI and AD [21, 26, 35, 38, 43]. Finally, one study found an association between larger white matter hyperintensities volumes and cortical hypoperfusion in AD [22]. Given that neurodegenerative and cerebrovascular pathology often coexist [75], this result speaks to the notion that ischaemic small vessel disease contributes to hypoperfusion in AD.

The only study on VCI in this review revealed both regional hypoperfusion and hyperperfusion in patients with small vessel and those with large vessel VCI [51]. Apart from brain perfusion, the study assessed neurovascular coupling. Brain regions with altered neurovascular coupling (i.e. altered perfusion or altered amplitude of low-frequency fluctuations in blood oxygen level-dependent functional MRI) were found to be localised at high levels of the cortical hierarchy, namely within the default mode network [51]. Nevertheless, given the heterogeneity of VCI, which can involve small or large vessels or the grey or white matter and can have a local or systemic cause [76], the aforementioned results should be complemented by those of future studies with larger samples and a longitudinal design.

Hypoperfused structures found to better help diagnosing patients with FTLD were the ACC and frontal regions [30]. Global brain hypoperfusion, with relative sparing of the temporal lobes, was found to be distinctive of DLB [23], even after correcting for partial volume effects, a pattern resembling the temporal lobe volume sparing typical of DLB. These findings represent a relevant clue for clinical diagnosis of both FTLD and DLB.

Although more studies on the differential diagnosis of NCD are warranted, pCASL was found to be helpful to discriminate patients with AD from those with FTLD (Fig. 2). As previously mentioned, a ROC curve generated for CBF measurements in the PCC revealed an optimal cut-off value of 46.3 ml/100 g/min to discriminate AD from FTLD in the early stage of disease progression [30]. In addition, compared to patients with AD, patients with DLB showed a more widespread pattern of cortical hypoperfusion, especially in the frontal, parietal and occipital cortices, as well as in the precunei and adjacent PCC [23].

Although brain perfusion could represent a biomarker of several psychiatric disorders [77], most studies included in this review did not find specific patterns of abnormal perfusion in SSD, BD or MDD [20, 27–29, 33, 36, 39, 40, 45]. The lack of findings suggests a predominance of other mechanisms underlying these conditions (e.g. abnormal neurotransmission, lack of synaptic plasticity and dysconnectivity) over microvascular or metabolic abnormalities. Such mechanisms are still poorly understood.

Perfusion abnormalities were reported in SSD with potential pathophysiological and clinical relevance [27, 29, 36, 39]. For instance, hyperperfusion in the temporal cortices and striata might mirror abnormally increased dopaminergic activity in these regions. Furthermore, hyperperfusion in the temporal cortices is consistent with the occurrence of auditory hallucinations in patients with SSD [78]. The association between increased perfusion in the left middle temporal gyrus and PANSS positive scores supports this idea [29]. Likewise, hypoperfusion in the prefrontal and anterior cingulate regions has been demonstrated, contributing to the well-established reduced neuropil hypothesis and to reduced effective connectivity found in the prefrontal regions and ACC of patients with schizophrenia [79]. It is also consistent with decreased dopaminergic activity in the prefrontal cortical pathway, possibly accounting for cognitive dysfunction and negative symptoms in these patients [80]. However, perfusion abnormalities in SSD were found to be more diffuse and topographically complex than the aforementioned, making them nearly impossible to detect by visual inspection in a clinical setting. In addition, most patients with SSD had chronic symptoms and were on antipsychotic medication. Given that cognitive impairment, grey matter loss and social isolation are expected to be more prominent in patients with chronic schizophrenia, these factors may confound interpretation and generalisation of the reported findings.

The studies on patients with BD or MDD were quite heterogeneous, particularly as patients with BD had distinct characteristics and medication schemes [33, 40, 45]. These differences could have prevented the identification of patterns of abnormal brain perfusion and hindered comparison of the reported results. Whereas three studies found discrepant brain perfusion abnormalities in patients with MDD, relative to HC subjects [28, 33, 45], another found no significant baseline differences [20]. However, a subgroup analysis of the latter study found remitting patients with MDD to have higher CBF in the parietal white matter, relative to non-remitting patients [20]. This result suggests that CBF, as assessed by pCASL, might be predictive of MDD remission, as has been demonstrated by the response of these patients to repetitive transcranial magnetic stimulation [81], a potential tool for individualised therapy.

Regarding studies assessing brain perfusion in samples of patients either with BD or MDD [33, 45], Chen et al. suggested that dysfunction of the neocerebellum might be involved in the pathophysiological processes of bipolar II disorder and MDD and that increased perfusion both in the neocerebellum and the left middle temporal gyrus could represent overlapping pathophysiology in BD and MDD [45]. The study by Zhao et al. contradicted these findings, showing hypoperfusion in the left dentate nucleus of the cerebellum in BD and MDD [33]. Curiously, a common theme in both studies is that each found abnormally perfused infratentorial structures in BD and MDD, indicating that microstructural and perfusion abnormalities in the prefrontal–thalamic–cerebellar circuit influence neurobiological processes of both disorders [33]. Nevertheless, it was not possible to determine whether the reported abnormalities represented a state or trait biomarker for these conditions, because all participants were experiencing a depressive episode [33, 45].

Although ASD and ADHD are both neurodevelopmental disorders according to the DSM-5, findings on ASD [25], ADHD [42], panic disorder [41] and alcohol use disorder [44] did not allow for any sort of generalisation due to only one study being identified for each condition. Additional drawbacks of the current review were the inability to include eligible studies on other prevalent psychiatric disorders [14] and the limited number of studies on SCD *plus* [43], DLB [23] and VCI [51]. Stringent application of the ASL technical guidelines (i.e. pCASL sequences with a single PLD time) [8] further reduced the sample of eligible studies. Given that sequences incorporating multi-PLD times are under development, studies reporting on their clinical contribution are warranted in the near future. Moreover, some studies on NCD included subjects with SCD as a control group [21–23, 35], instead of HC subjects. Although this might be regarded as a limitation, including such populations might better reflect a typical clinical scenario. Finally, in the current review, only one study reported results based on a longitudinal design [20].

Nineteen (58%) of the included studies [20–23, 25, 26, 30, 31, 33–38, 43, 44, 46, 48, 50] reported absolute CBF measurements (in mL/100 g/min). Six of these [22, 25, 34, 36, 37, 48] just reported global brain or grey matter CBF. The remaining 14 (42%) studies [24, 27–29, 32, 39–42, 45, 47, 49, 51, 52] reported regions of abnormal brain perfusion. The reported findings were demonstrated at the between-subject level in all studies [20–52]. Only one study [30] also presented an exemplary illustration on the utility of pCASL for clinical diagnosis at the within-subject level (Fig. 2).

Clinical heterogeneity (i.e. differences in patient characteristics) across samples may explain the diversity of results reported by the studies reviewed herein, particularly demographic factors (age, sex and education), concurrent medication and comorbidities. Likewise, the significance of abnormal global or regional brain perfusion depends on the understanding of physiological correlates at the neurovascular level. Nevertheless, our results support the use of biomarkers for the diagnosis of NCD and other mental disorders. Molecular, biological (e.g. liquid biopsy) and imaging-based (e.g. morphometric, perfusion-based, quantitative susceptibility mapping [82]) biomarkers are of particular interest. Prior reviews [4, 83, 84], as well as current guidance on ASL in the routine clinical setting [5], also support pCASL as being a reliable biomarker for the detection and diagnosis of NCD and its incipient forms. Implementation in clinical practice requires caution, however. For instance, the effect of perfusion modifiers (e.g. caffeine, age and blood gases) must be considered for diagnostic accuracy. Some of these factors alter CBF measurements in a similar way as pathological conditions and, therefore, can hinder accurate diagnoses [85].

In conclusion, brain perfusion abnormalities were found to help diagnose most NCD. Abnormalities reported in VCI and other mental disorders were heterogeneous and not generalisable, but some promising reports indicate an increasing role for the assessment of brain perfusion by pCASL, especially if this advanced MRI technique is combined with evaluation of neurovascular coupling or with functional and effective brain connectivity analyses.

### Supplementary Information

Below is the link to the electronic supplementary material.

Supplementary file1 (DOCX 45 KB)
