# Supplementary material for: Arterial spin labelling magnetic resonance imaging and perfusion patterns in neurocognitive and other mental disorders: a systematic review
Source: Neuroradiology. 2024 Mar 27;66(7):1065–81. doi: 10.1007/s00234-024-03323-0 (PMC11150205; doi:10.1007/s00234-024-03323-0)
Supplement: Supplementary file 1 — Supplementary file1 (DOCX 45 KB) [file 234_2024_3323_MOESM1_ESM.docx]

**Supplementary Table 1. Main characteristics of the included studies, including the post-labelling delay (PLD) time of the applied pseudo-continuous arterial spin labelling (pCASL) sequence.**

| **Study** | **Sample** | **n (men)** | **Age (years)** | **PLD** |
| --- | --- | --- | --- | --- |
| Järnum et al. (2011) [20] | 23 patients with MDD^†^;  26 HC subjects^†^ | 7 13 | (43; SD = 9.9) (42; SD = 13.7) | 1500 ms |
| Binnewijzend et al. (2013) [21] | 71 patients with AD^ǂ^; 31 patients with MCI ^ǂ^;  70 SCD subjects^ǂ^ | 32 18 43 | (65; SD = 7.0) (65; SD = 8.0) (60; SD = 9.0) | 2000 ms |
| Benedictus et al. (2014) [22] | 129 patients with AD^ǂ^; 61 SCD subjects^ǂ^ | 60 35 | (66; SD = 7.0) (64; SD = 5.0) | 2000 ms |
| Binnewijzend et al. (2014) [23] | 22 patients with bvFTD; 14 patients with DLB; 48 patients with AD ^ǂ^; 50 SCD subjects^ǂ^ | 14 12 22 32 | (63; SD = 7.0) (66; SD = 8.0) (65; SD = 7.0) (62; SD = 6.0) | 2000 ms |
| Ding et al. (2014) [24] | 24 patients with AD; 17 patients with aMCI; 21 HC subjects | 5 6 8 | (75; SD = 6.7) (71; SD = 7.6) (70; SD = 5.9) | 1500 ms |
| Jann et al. (2015) [25] | 17 patients with ASD; 22 HC subjects | 13 19 | (14; SD = 2.0) (13; SD = 3.6) | 1000 ms |
| Liu et al. (2015) [26] | 16 patients with AD; 19 HC subjects | 6 5 | (75; SD = 6.9) (70; SD = 8.1) | 1500 ms |
| Zhu et al. (2015) [27] | 100 patients with SSD; 94 HC subjects | 57 44 | (34; SD = 8.6) (33; SD = 10.4) | 2025 ms |
| Kaichi et al. (2016) [28] | 53 patients with MDD; 36 HC subjects | 27 17 | (42; SD = 10.9) (40; SD = 11.6) | 1525 ms |
| Ma et al. (2016) [29] | 95 patients with SSD; 99 HC subjects | 51 43 | (34; SD = N/A) (34; SD = N/A) | 2025 ms |
| Steketee et al. (2016) [30] | 13 patients with presenile AD; 19 patients with presenile FTLD*; 25 HC subjects; | 8 11 13 | (62; SD = 5.5) (63; SD = 4.5) (61; SD = 5.9) | 1525 ms |
| Steketee et al. (2016) [31] | 11 patients with bvFTD; 7 patients with phFTD; 20 HC subjects | 5 7 20 | (63; SD = N/A) (61; SD = N/A) (64; SD = N/A) | 1525 ms |
| Steketee et al. (2016) [32] | 11 patients with AD; 9 patients with bvFTD; 18 HC subjects | 8 4 8 | (62; SD = 5.0) (62; SD = 5.7) (60; SD = 6.7) | 1525 ms |
| Zhao et al. (2016) [33] | 35 patients with BD; 30 patients with UD; 45 HC subjects | 18 13 22 | (30; SD = 10.1) (34; SD = 9.3) (32; SD = 11.7) | 1525 ms |
| Dolui et al. (2017) [34] | 24 patients with MCI; 22 HC subjects | 17 5 | (74; SD = 7.4) (71; SD = 7.0) | 1500 ms |
| Leeuwis et al. (2017) [35] | 161 patients with AD ^ǂ^; 95 patients with MCI ^ǂ^; 143 SCD subjects^ǂ^ | 73 62 80 | (66; SD = 7.0) (65; SD = 7.3) (60; SD = 8.7) | 2000 ms |
| Zhu et al. (2017) [36] | 89 patients with SSD; 90 HC subjects | 49 42 | (34; SD = 7.8) (33; SD = 10.2) | 2025 ms |
| Anazodo et al. (2018) [37] | 10 patients with FTLD^††^; 10 HC subjects | 5 4 | (66; SD = 6.6) (67; SD = 6.6) | 1500 ms |
| Huang et al. (2018) [38] | 50 patients with AD  30 HC subjects | 17 12 | (73; SD = 8.4) (71; SD = 8.1) | 1525 ms |
| Kim et al. (2019) [39] | 12 patients with SSD; 11 HC subjects | 7 8 | (45; SD = 12.1) (41; SD = 12.9) | 1525 ms |
| Dai et al. (2020) [40] | 19 patients with BD; 26 HC subjects | 10 12 | (34; SD = 9.7) (31; SD = 10.9) | 1800 ms |
| Ni et al. (2020) [41] | 26 patients with panic disorder; 27 HC subjects | 12 12 | (35; SD = 6.5) (35; SD = 6.1) | 2025 ms |
| Tan et al. (2020) [42] | 69 patients with ADHD; 69 HC subjects | 69 69 | (27; SD = 3.9) (26; SD = 3.9) | 1525 ms |
| Yang et al. (2021) [43] | 31 patients with aMCI; 27 subjects with SCD plus; 33 HC subjects | 12 12 13 | (69; SD = 6.9) (68; SD = 7.0) (67; SD = 6.1) | 1500 ms |
| Butcher et al. (2022) [44] | 15 patients with AUD; 22 HC subjects | 9 9 | (37; SD = 11.2) (36; SD = 11.9) | 1800 ms |
| Chen et al. (2022) [45] | 88 patients with BD type II; 95 patients with MDD; 96 HC subjects | 41 32 46 | (25; SD = 7.7) (28; SD = 9.3) (28; SD = 7.7) | 1525 ms |
| Ssali et al. (2022) [46] | 6 patients with FTLD^†††^; 13 HC subjects | N/A 8 | N/A (64; SD = 9.9) | 2000 ms |
| Gao et al. (2023) [47] | 74 patients with AD; 74 patients with aMCI; 74 HC subjects | 33 29 31 | (65; SD = N/A) (65; SD = N/A) (63; SD = N/A) | 2025 ms |
| Camargo et al. (2023) [48] | 16 patients with AD; 25 patients with MCI; 40 HC subjects | 11 18 22 | (73; SD = 7.8) (75; SD = 8.6) (75; SD = 7.2) | 2000 ms |
| Dong et al. (2023) [49] | 42 patients with AD; 49 HC subjects | 11 20 | (65; SD = 8.5) (67; SD = 6.0) | 2025 ms |
| Mao et al. (2023) [50] | 66 patients with AD; 21 patients with MCI; 26 patients with FTLD; 21 HC subjects | 21 11 15 6 | (66; SD = 10.2) (67; SD = 7.5) (62; SD = 9.1) (64; SD = 6.5) | 2025 ms |
| Ruan et al. (2023) [51] | 38 patients with SVCI; 34 patients with PSCI; 43 HC subjects | 30 27 33 | (61; SD = 8.4) (61; SD = 8.7) (58; SD = 4.4) | 2025 ms |
| Zhu et al. (2023) [52] | 89 patients with AD; 74 patients with aMCI; 66 HC subjects | 37 31 28 | (67; SD = N/A) (64; SD = N/A) (63; SD = N/A) | 2025 ms |

AD: Alzheimer's disease; ADHD: attention‐deficit/hyperactivity disorder; aMCI: amnestic mild cognitive impairment; ASD: autism spectrum disorder; AUD: alcohol use disorder; BD: bipolar disorder; bvFTD: behavioural variant of frontotemporal dementia; DLB: dementia with Lewy Bodies; FTLD: frontotemporal lobar degeneration; HC: healthy control; MCI: mild cognitive impairment; MDD: major depressive disorder; ms: milliseconds; n: number; N/A: not available; phFTD: phenocopy frontotemporal dementia; PLD: post-labelling delay; PNFA: primary progressive nonfluent aphasia; PSCI: post-stroke cognitive impairment ; SCD: subjective cognitive decline; SD: semantic dementia; SSD: schizophrenia spectrum disorder; SVCI: small vessel disease cognitive impairment ; UD: unipolar depression; ^†^Reported CBF values refer to 20 patients with MDD and 20 healthy control subjects at baseline; ^ǂ^Potential overlap of included subjects, since they were retrieved from the same memory clinic; *Includes both bvFTD patients and FTLD underlying primary progressive aphasia; ^††^ Of the 10 FTLD patients, seven were diagnosed with bvFTD, one patient met criteria for bvFTD and non-fluent primary progressive aphasia, one patient was diagnosed with semantic variant/primary progressive aphasia with behavioural features, and one patient was diagnosed with possible bvFTD; ^†††^Of the 6 FTLD patients, two were diagnosed with bvFTD, two with SD, and two with PNFA. Age data are reported as mean and standard deviation [SD])
